# Depressive symptoms predict longitudinal changes of chronic inflammation at the transition to adulthood

**DOI:** 10.3389/fimmu.2022.1036739

**Published:** 2023-01-04

**Authors:** Shuang Zhai, Yang Qu, Dan Zhang, Tingting Li, Yang Xie, Xiaoyan Wu, Liwei Zou, Yajuan Yang, Fangbiao Tao, Shuman Tao

**Affiliations:** ^1^ Department of Maternal, Child and Adolescent Health, School of Public Health, Anhui Medical University, Hefei, China; ^2^ Key Laboratory of Population Health Across Life Cycle, Ministry of Education of the People's Republic of China, Hefei, China; ^3^ Key Laboratory of Study on Abnormal Gametes and Reproductive Tract, National Health Commission of the People's Republic of China, Hefei, China; ^4^ Department of Ophthalmology, The Second Hospital of Anhui Medical University, Hefei, China

**Keywords:** Depressive symptoms, dose-response relationship, follow-up study, inflammatory biomarkers, young adults

## Abstract

**Background:**

Inflammation is closely related to poor mental and physical health, including depressive symptoms and its specific symptoms. To reveal the linear and nonlinear relationships between depressive symptoms and chronic inflammation levels, and perform further analysis of the associations between symptom-specificity of depressive symptoms and inflammation among young adults by using a prospective design.

**Methods:**

In this longitudinal study, we examined college students recruited from two universities in China, who were examined at baseline and 2-years follow-up. Depressive symptoms were measured by applying the Patient Health Questionnaire 9 (PHQ-9) at baseline. Plasma levels of four inflammatory biomarkers, including interleukin-6 (IL-6), interleukin-1β (IL-1β), tumor necrosis factor-α (TNF-α), and C reactive protein (CRP) were assayed at baseline and 2-year follow-up. In addition to the conventional generalized linear models, as well as restricted cubic splines were innovatively used to analyze the cross-sectional and longitudinal nonlinear relationships between depressive symptoms and inflammatory biomarkers.

**Results:**

Generalized linear model analysis revealed that there were no statistical associations between depressive symptoms and any inflammatory biomarker levels. The results of the restricted cubic spline demonstrated a U-shaped nonlinear association between depressive symptoms and ΔIL-1β or ΔTNF-α (changes in baseline and 2-year follow-up), but these associations disappeared after adjusting the confounders. Symptom-specificity of depressive symptoms such as sleeping problems and suicidal ideation were associated with lower IL-1β at baseline or changes in IL-1β levels. Sleeping problems and psychomotor changes at baseline were associated with higher CRP at 2-year follow-up. Suicidal ideation at baseline was associated with changes in TNF-α levels.

**Conclusion:**

Our findings suggested that symptom-specificity of depressive symptoms was associated with inflammation during a 2-year follow-up at the transition to adulthood. Simultaneously, more research is warranted to seek the directionality of depressive symptoms and chronic inflammation.

## 1 Introduction

The inflammatory response is a basic part of the body’s innate immune mechanism to defend itself from detrimental stimuli, even though it is often considered a response to physical threats (1). Activation of the inflammatory pathway also affects the endocrine system, including stimulation of the hypothalamic-pituitary-adrenal axis (HPA), thereby causing down-regulation of glucocorticoid receptors, and decreased hormone response to inflammation, and subsequent inflammatory biomarker levels changed (2). Furthermore, common pro-inflammatory inflammatory biomarkers such as IL-6, IL-1β, TNF-α, CRP, etc. (3–6), were presently recognized. Regardless, systemic inflammation can damage physical health, such as cardiovascular disease and chronic disease. Moreover, there is evidence that it is also strongly related to poor mental health, including major depressive disorders (7). Growing evidence recommends that inflammatory processes are connected with the pathophysiology of depressive symptoms and play a role in the etiology and course of mood disorders. Nonetheless, the precise duty of inflammation in the etiology of depression remains unknown (8, 9). Moreover, inflammation is associated with some but not all depressive symptoms, such as low mood and anhedonia and somatic/neurovegetative symptoms of fatigue, altered sleep, and appetite changes (10). Therefore, a symptom-based treatment may provide insights into the mechanisms of inflammation-related depression.

In a cross-sectional study, individuals with depression had higher levels of IL-6 than healthy participants and frequently exhibited altered peripheral inflammatory profiles (11). Another cross-sectional study with adult males revealed significantly higher serum TNF-α levels in the depressive symptoms group than in the non-depressive symptoms group (12). It should be pointed out that there is a review that argues that the link between depression and inflammation may be bidirectional (13). A previous prospective study found a weaker bidirectional association between CRP and depressive symptoms in the elderly (14). Data from the UK Biobank manifested that higher inflammatory biomarkers levels were associated with an increased risk of depressive symptoms (15). A recent study reported depression risk score at 12 years old was positively linked to pro-inflammatory biomarkers levels 6 years later in the British (16). The incidence of depressive symptoms in children and adolescents is increasing sharply and the first onset of depression is often in adolescence or early adulthood (17). This is also an important window for identifying modifiable behavioral risk factors to prevent and control depression apparently (18). Nevertheless, most previous studies focus on middle-aged or elderly adults, there is a lack of prospective studies on depressive symptoms that causes elevated levels of inflammation at the transition to adulthood, which is an important phase in the development of depressive symptoms.

College students are in the critical stage of transition from adolescence to adulthood and healthy physical and mental development (19). Owing to most of the previous research participants being adults, there were few related studies on college students and the results were inconsistent obviously. Most research has indicated a linear positive correlation with higher levels of pro-inflammatory biomarkers in depressed individuals (11, 12, 20). On the contrary, some research unexpectedly displayed pro-inflammatory biomarkers were negatively correlated with depression (15). Additionally, rare studies have explored the nonlinear relationship between depression and chronic inflammation utilizing restricted cubic splines compared to the more traditional analyses in most studies. To address the indispensable evidence gap, this study was the first to focus on the linear and nonlinear association between depression and chronic inflammation in Chinese college students based on a longitudinal study.

## 2 Methods

### 2.1 Study population

The data came from the ongoing College Student Behavior and Health Cohort Study, which aimed to assess college students’ health behavior and mental health in China. Based on the convenient sampling, a medical college and a comprehensive normal college were selected in Hefei City, Anhui Province, and Shangrao City, Jiangxi Province to recruit the freshman students. Then all freshmen from two faculty of each university were selected by cluster random sampling. A prospective cohort study with baseline and 4-wave follow-up at 6-month intervals in 2 years was conducted from freshman year to junior year. The baseline survey was conducted from April to May 2019. A total of 723 questionnaires and blood samples were collected at baseline. Electronic questionnaires were used, which were expected to take 15 to 20 minutes to fill out on a smartphone. Due to uncontrollable factors such as the COVID-19 epidemic and the health status of students, 248 questionnaires and blood samples were collected both at the baseline and 2-year follow-up, and no one was infected by the COVID-19 virus and designated as having experienced definite psychotic symptoms. The authors assert that all procedures used in this study comply with the ethical standards of the relevant national and institutional committees on human experimentation and with the Helsinki Declaration of 1975, as revised in 2008. The research protocol was approved by the Ethics Committee of Anhui Medical University (No. 20170291), and all participating students obtained written informed consent before completing the survey.

### 2.2 Depressive symptoms and symptom-specificity

The Patient Health Questionnaire (PHQ)-9 was applied to evaluate the symptoms of depression at baseline. Total scores for PHQ-9 range from 0 to 27 by summarizing scores of nine screening items with higher scores predicting more severe depressive symptoms. Depressive symptoms were defined as 5 points, 0-4 were divided into the no depressive symptoms group, and ≥ 5 were divided into the depressive symptoms group (21). In the present study, Cronbach’s alpha coefficient was 0.917. Compared to clinically diagnosed depression, more depressive symptoms can be captured through self-report, which may lead to an overestimation of the prevalence of depression. However, there was also evidence that self-report measures of depressive symptoms were superior to clinician ratings, since rating scales used routinely in clinical practice may be impractical (22). Otherwise, PHQ-9 is recommended for research and clinical evaluation by the American Psychiatric Association and uses the DSM-IV diagnostic criteria to assess depressive symptomatology, such as sleep, concentration, energy problems, low self-esteem, anhedonia, etc. (23).

Symptom-specificity of depressive symptoms was recorded into “psychological symptoms” and “somatic symptoms” based on the reported PHQ-9 symptoms, including anhedonia, depressed mood, sleeping problems, fatigue, appetite changes, feelings of inadequacy, cognitive problems, psychomotor changes, and suicidal ideation.

### 2.3 Inflammatory biomarkers

Blood samples were collected at baseline and 2-year follow-up. From 6:00 to 8:00 in the morning, vacuum blood collection tubes (anticoagulation tubes) were used to take 5 mL of fasting venous blood for collection by medical professionals. Participants were instructed to avoid caffeinated foods (chocolate, coffee, tea, cola), alcohol, anti-inflammatory drugs such as aspirin and ibuprofen, and strenuous physical activity the day before venous blood collection. Blood samples were centrifuged at 3 000 rpm for 10 minutes within 2 hours. The upper plasma samples were split into 500 μL cryovials and stored in a -80°C refrigerator. The levels of inflammatory cytokine IL-6, IL-1β, and TNF-α in the supernatant were detected by a liquid-phase protein suspension chip detection instrument. The coefficient of variation of all analytes was less than 5%, and the coefficient of variation between batches of IL-6, IL-1β, and TNF-α was less than 20%, 15%, and 15%, respectively. The detection limit concentration values for IL-6, IL-1β, and TNF-α were 0.11 pg/mL, 0.14 pg/mL, and 0.16 pg/mL, respectively. The level of CRP was measured by immunoturbidimetry using the serum on the day the blood samples were collected.

### 2.4 Covariates

Due to the possible confounding influence of individual and family characteristics, we considered gender (males/females), age, residential area (rural/urban), any siblings (yes/no), self-reported family economy (low/medium or high), self-rated health condition (low/medium or high), a parental education level (primary school and below/middle school/senior high school and above) as covariates. Besides, we also considered some confounding factors that may affect the level of inflammation, including body mass index (BMI) calculated by height and weight, and risk health behaviors such as cigarette use (yes/no) and alcohol use (yes/no), which were classified by answering two modified questions based on the Youth Risk Behaviour Surveillance System questionnaire (24).

### 2.5 Statistical analyses

Nominal data are deliberated as frequency and percentage, and continuous data with Gaussian distribution are expressed as mean and standard deviation (SD). Group differences were tested with Chi-Squared, Kruskal-Wallis tests, or two independent sample t-tests. All statistical tests were two-sided and significance was set at *P*<0.05.

A generalized linear model and restricted cubic spline model were realized to examine the linear and nonlinear associations of PHQ-9 scores with IL-1β, IL-6, TNF-α, and CRP levels. Changes between inflammatory biomarker levels at baseline and 2-year follow-up were quantified by calculation difference scores (Δ=follow-up minus baseline). Since the levels of IL-1β, IL-6, TNF-α, and CRP at baseline and follow-up all skewed distribution, logarithmic transformation was performed. Restricted cubic splines were applied to seek a possible nonlinear dependency between the measures. Restricted cubic splines consisted of polynomial functions that fit well with nonlinear relationships. The choice of knots depended on the setting of the research objectives. In the great majority of studies, 3 to 5 knots were generally selected. According to statistical test results, the choice of 3 knots was smoother than 5 knots and can better test the linear deviation. Three knots were pre-specified in the 5th, 50th, and 95th percentiles, and one component of the spline function was generated (25–27). Controlling factors included residential area, any siblings, and parental education level. Further, we also explored the association between the symptom-specificity of depressive symptoms and inflammatory biomarkers by generalized linear models.

Sensitivity analyses were performed by using Chi-Squared tests (categorical variables) or t-tests (continuous variables) to compare the difference in characteristics of participants who were included in the analysis and those who were excluded because of dropping out.

The construction of the cubic spline model used the rms package in the software R version 4.1.2, all other statistical analyzes were performed using SPSS version 23.0.

## 3 Results

### 3.1 Characteristics of the study population

As shown in **Table 1**, 723 college students aged 16 to 26 (M=18.68, SD=0.99) completed the online questionnaires, of which 238 (32.9%) were males. Blood sample collection was completed both at baseline and follow-up. At baseline, the prevalence rate of depressive symptoms was 40.1%.

**Table 1:** Characteristics of the study sample (n=723).

Variables	Total sample [*n* (%)]	Depressive symptoms at baseline (n=290)		
		*n* (%)	*χ* ^2^/*t* value	*P* value
Gender			0.008	0.931
Male	238 (32.9)	96 (40.3)		
Female	485 (67.1)	194 (40.0)		
Age, mean ± SD	18.68 ± 0.99	18.73 ± 0.85	-1.219	0.223
BMI, mean ± SD	20.73 ± 2.59	20.72 ± 2.60	0.090	0.928
Residential area			9.128	0.003
Rural	407 (56.3)	183 (45.0)		
Urban	316 (43.7)	107 (33.9)		
Any siblings			2.562	0.109
No	185 (25.6)	65 (35.1)		
Yes	538 (74.4)	225 (41.8)		
Self-reported family economy			6.157	0.046
Low	160 (22.1)	77 (48.1)		
Medium	525 (72.6)	201 (38.3)		
High	38 (5.3)	12 (31.6)		
Self-rated health condition			29.253	<0.001
Low	23 (3.2)	17 (73.9)		
Medium	265 (36.7)	130 (49.1)		
High	435 (60.1)	143 (32.9)		
Father’s educational level			6.836	0.033
Primary school and below	160 (22.1)	74 (46.3)		
Middle school	350 (48.4)	145 (41.4)		
Senior high school and above	213 (29.5)	71 (33.3)		
Mother’s educational level			6.068	0.048
Primary school and below	322 (44.5)	142 (44.1)		
Middle school	249 (34.5)	99 (39.8)		
Senior high school and above	152 (21.0)	49 (32.2)		
Cigarette use			4.765	0.029
No	678 (93.8)	265 (39.1)		
Yes	45 (6.2)	25 (55.6)		
Alcohol use			6.135	0.013
No	567 (78.4)	214 (37.7)		
Yes	156 (21.6)	76 (48.7)		
Baseline, mean ± SD (n=723)				
IL-1β	0.32 ± 0.26	0.31 ± 0.27	0.722	0.471
IL-6	0.72 ± 0.32	0.72 ± 0.32	0.298	0.766
TNF-α	0.77 ± 0.21	0.76 ± 0.21	0.437	0.662
CRP	-0.26 ± 0.50	-0.28 ± 0.53	0.748	0.454
2-year follow-up, mean ± SD (n=248)				
F-IL-1β^a^	0.35 ± 0.23	0.36 ± 0.22	-0.440	0.661
F-IL-6	-0.52 ± 0.85	-0.58 ± 0.87	1.096	0.274
F-TNF-α	0.38 ± 0.22	0.39 ± 0.22	0.328	0.668
F-CRP	-0.04 ± 0.36	-0.02 ± 0.35	-0.949	0.343
Follow-up minus baseline, mean ± SD (n=248)				
ΔIL-1β^b^	-0.03 ± 0.37	0.00 ± 0.39	-1.313	0.190
ΔIL-6	-1.25 ± 0.80	-1.31 ± 0.80	0.956	0.340
ΔTNF-α	-0.44 ± 0.32	-0.41 ± 0.34	-1.268	0.206
ΔCRP	0.36 ± 0.54	0.39 ± 0.53	-0.769	0.442

SD, standard deviation; BMI, body mass index; IL-1β, interleukin-1β; IL-6, interleukin-6; TNF-α, tumor necrosis factor-α; CRP, C reactive protein.

a F represented 2-year follow-up with baseline.

b Δ represented changes in inflammatory biomarkers levels between 2-year follow-up and baseline.

Students from rural areas suffered from a higher risk of depressive symptoms compared to those from urban areas (*P*=0.003). Those students who reported lower family economy (*P*=0.046) or self-rated health conditions (*P*<0.001) were more likely to report depressive symptoms. The rates of depressive symptoms were higher in respondents who reported lower parental educational levels (*P*<0.05). Compared with non-drinkers or non-smokers, drinkers or smokers were more likely to report depressive symptoms. Gender, age, BMI, and any siblings did not differ between the participants with depressive symptoms and healthy participants at baseline.

Inflammatory biomarker levels at baseline (IL-1β, IL-6, TNF-α, and CRP), 2-year follow-up (FIL-1β, FIL-6, FTNF-α, and FCRP), or the changes between follow-up and baseline (ΔIL-1β, ΔIL-6, ΔTNF-α, and ΔCRP) were not statistically different between participants with depressive symptoms and healthy participants at baseline.

### 3.2 Associations between baseline PHQ-9 scores and inflammatory biomarkers

**Tables 2**–**4** depict the linear relationships between baseline PHQ-9 scores and inflammatory biomarker levels at baseline or 2-year follow-up, as well as the changes in inflammatory biomarker levels between follow-up and baseline. In the generalized linear models, individuals with higher PHQ-9 total scores at baseline were more likely to have lower CRP at follow-up and changes in CRP between follow-up and baseline. After controlling for residential area, self-reported family economy, self-rated health condition, father’s education level, mother’s education level, cigarette use, and alcohol use, the results remained no statistical significance.

**Table 2:** Generalized linear models of the associations between PHQ-9 total scores and inflammatory biomarkers at baseline (n=723).

Variables	IL-1β		IL-6		TNF-α		CRP	
	*B* (95% *CI*)	*P* value	*B* (95% *CI*)	*P* value	*B* (95% *CI*)	*P* value	*B* (95% *CI*)	*P* value
Crude model								
PHQ-9 total scores	-0.005 (-0.014, 0.003)	0.195	-0.006 (-0.016, 0.004)	0.246	-0.005 (-0.012, 0.001)	0.078	-0.002 (-0.010,0.132)	0.717
Adjusted model								
PHQ-9 total scores	0.001 (-0.004, 0.005)	0.815	0.001 (-0.005, 0.006)	0.761	0.001 (-0.004, 0.004)	0.948	-0.001 (-0.010,0.008)	0.838
Residential area								
Rural	0.032 (-0.013, 0.077)	0.165	0.001 (-0.055,0.055)	0.991	0.015 (-0.022,0.051)	0.434	-0.089 (-0.176,-0.001)	0.047
Urban	Ref.		Ref.		Ref.		Ref.	
Self-reported family economy								
Low	-0.039 (-0.134,0.057)	0.430	-0.076 (-0.194,0.041)	0.201	0.001 (-0.078,0.077)	0.991	-0.245 (-0.431, -0.059)	0.010
Medium	-0.029 (-0.115,0.056)	0.499	-0.024 (-0.128,0.081)	0.656	-0.013 (-0.081,0.056)	0.721	-0.257 (-0.423, -0.091)	0.002
High	Ref.		Ref.		Ref.		Ref.	
Self-rated health condition								
Low	0.091 (-0.016,0.199)	0.096	0.015 (-0.117,0.146)	0.827	0.094 (0.007,0.181)	0.034	-0.031 (-0.241,0.178)	0.768
Medium	0.064 (0.024,0.105)	0.002	0.055 (0.006,0.104)	0.029	0.046 (0.014,0.079)	0.005	0.022 (-0.056,0.100)	0.576
High	Ref.		Ref.		Ref.		Ref.	
Father’s educational level								
Primary school and below	-0.094 (-0.156,-0.031)	0.003	-0.087 (-0.163,-0.011)	0.026	-0.069 (-0.119, -0.018)	0.008	0.078 (-0.043,0.199)	0.208
Middle school	-0.050 (-0.102,0.002)	0.060	-0.038 (-0.101,0.025)	0.239	-0.047 (-0.089, -0.005)	0.028	0.082 (-0.019,0.183)	0.110
Senior high school and above	Ref.		Ref.		Ref.		Ref.	
Mother’s educational level								
Primary school and below	0.003 (-0.062,0.067)	0.936	-0.018 (-0.097,0.060)	0.648	0.010 (-0.042,0.062)	0.705	-0.007 (-0.132,0.117)	0.906
Middle school	0.011 (-0.047,0.070)	0.703	0.006 (-0.066,0.078)	0.864	0.030 (-0.017,0.078)	0.211	-0.020 (-0.134,0.094)	0.733
Senior high school and above	Ref.		Ref.		Ref.		Ref.	
Cigarette use	-0.118 (-0.200, -0.036)	0.005	-0.124 (-0.224,-0.024)	0.015	-0.064 (-0.130,0.002)	0.059	-0.019 (-0.179,0.140)	0.811
Alcohol use	-0.033 (-0.080,0.014)	0.171	-0.057 (-0.115,0.001)	0.055	-0.026 (-0.064,0.012)	0.182	-0.006 (-0.099,0.086)	0.838

Inflammatory cytokines were log-transformed before analysis; the crude model was not adjusted by any variables, the adjusted model was adjusted by residential area, self-reported family economy, self-rated health condition, father’s education level, mother’s education level, cigarette use and alcohol use.

B, regression coefficient; CI, confidence interval; IL-1β, interleukin-1β; IL-6, interleukin-6; TNF-α, tumor necrosis factor-α; CRP, C reactive protein.

**Table 3:** Generalized linear models of the associations between PHQ-9 total scores at baseline and inflammatory biomarkers at follow-up (n=248).

Variables	FIL-1β^a^		FIL-6		FTNF-α		FCRP	
	*B* (95% *CI*)	*P* value	*B* (95% *CI*)	*P* value	*B* (95% *CI*)	*P* value	*B* (95% *CI*)	*P* value
Crude model								
PHQ-9 total scores	0.003 (-0.004, 0.009)	0.425	-0.015 (-0.041, 0.009)	0.205	0.002 (-0.004, 0.009)	0.482	0.103 (0.088,0.118)	<0.001
Adjusted model								
PHQ-9 total scores	0.005 (-0.002,0.012)	0.174	-0.008 (-0.033,0.018)	0.544	-0.001 (-0.007,0.006)	0.798	0.003 (-0.008,0.014)	0.591
Residential area								
Rural	0.004 (-0.064,0.072)	0.903	0.091 (-0.159,0.341)	0.476	-0.012 (-0.075,0.051)	0.703	-0.027 (-0.134,0.081)	0.627
Urban	Ref.		Ref.		Ref.		Ref.	
Self-reported family economy								
Low	-0.092 (-0.257,0.073)	0.275	0.259 (-0.347,0.865)	0.402	-0.022 (-0.175,0.130)	0.774	-0.008 (-0.269,0.253)	0.952
Medium	-0.063 (-0.214,0.089)	0.418	0.362 (-0.195,0.918)	0.203	-0.015 (-0.155,0.126)	0.838	0.021 (-0.218,0.261)	0.861
High	Ref.		Ref.		Ref.		Ref.	
Self-rated health condition								
Low	-0.074 (-0.257,0.110)	0.430	0.374 (-0.299,1.047)	0.276	-0.153 (-0.323,0.017)	0.077	-0.124 (-0.414,0.166)	0.403
Medium	0.004 (-0054,0.062)	0.894	0.219 (0.005,0.433)	0.045	-0.006 (-0.060,0.048)	0.819	-0.026 (-0.118,0.066)	0.578
High	Ref.		Ref.		Ref.		Ref.	
Father’s educational level								
Primary school and below	-0.122 (-0.216,-0.028)	0.011	-0.422 (-0.768, -0.077)	0.017	0.033 (-0.054,0.120)	0.461	0.064 (-0.085,0.213)	0.400
Middle school	-0.103 (-0.180, -0.026)	0.009	-0.327 (-0.610, -0.044)	0.023	-0.028 (-0.099,0.044)	0.447	0.017 (-0.105,0.139)	0.782
Senior high school and above	Ref.		Ref.		Ref.		Ref.	
Mother’s educational level								
Primary school and below	0.060 (-0.037,0.156)	0.226	-0.046 (-0.400,0.309)	0.800	0.073 (-0016,0.162)	0.109	0.030 (-0.123,0.183)	0.701
Middle school	0.048 (-0.043,0.139)	0.299	-0.029 (-0.363,0.304)	0.863	0.039 (-0.045,0.123)	0.368	0.033 (-0.110,0.177)	0.649
Senior high school and above	Ref.		Ref.		Ref.		Ref.	
Cigarette use	-0.009 (-0.122,0.104)	0.874	-0.347 (-0.761,0.066)	0.100	0.149 (0.045,0.253)	0.108	0.114 (-0.064,0.292)	0.211
Alcohol use	0.037 (-0.031,0.105)	0.290	-0.080 (-0.330,0.171)	0.533	0.052 (-0.011,0.115)	0.108	0.093 (-0.015,0.201)	0.092

Inflammatory cytokines were log-transformed before analysis; the crude model was not adjusted by any variables, the adjusted model was adjusted by residential area, self-reported family economy, self-rated health condition, father’s education level, mother’s education level, cigarette use and alcohol use.

B, regression coefficient; CI, confidence interval; IL-1β, interleukin-1β; IL-6, interleukin-6; TNF-α, tumor necrosis factor-α; CRP, C reactive protein.

a F represented 2-year follow-up with baseline.

**Table 4:** Generalized linear models of the associations between PHQ-9 total scores at baseline and changes in inflammatory biomarkers between follow-up and baseline (n=248).

Variables	ΔIL-1β^a^		ΔIL-6		ΔTNF-α		ΔCRP	
	*B* (95% *CI*)	*P* value	*B* (95% *CI*)	*P* value	*B* (95% *CI*)	*P* value	*B* (95% *CI*)	*P* value
Crude model								
PHQ-9 total scores	0.008 (-0.003,0.019)	0.137	-0.010 (-0.034,0.013)	0.391	0.008 (-0.002,0.017)	0.109	0.136 (0.056,0.216)	0.001
Adjusted model								
PHQ-9 total scores	0.008 (-0.003,0.019)	0.155	-0.005 (-0.029,0.020)	0.714	0.004 (-0.006,0.013)	0.448	0.005 (-0.012,0.021)	0.561
Residential area								
Rural	-0.005 (-0.115,0.105)	0.930	0.078 (-0.161,0.317)	0.521	-0.006 (-0.099,0.088)	0.907	0.141 (-0.021,0.303)	0.089
Urban	Ref.		Ref.		Ref.		Ref.	
Self-reported family economy								
Low	-0.099 (-0.366,0.168)	0.468	0.389 (-0.191,0.968)	0.189	-0.019 (-0.245,0.207)	0.870	0.351 (-0.043,0.744)	0.081
Medium	-0.094 (-0.339,0.152)	0.455	0.385 (-0.147,0.917)	0.156	-0.047 (-0.254,0.161)	0.660	0.454 (0.092,0.815)	0.014
High	Ref.		Ref.		Ref.		Ref.	
Self-rated health condition								
Low	-0.210 (-0.507,0.087)	0.166	0.332 (-0.312,0.976)	0.312	-0.249 (-0.500,0.002)	0.052	0.252 (-0.185,0.689)	0.259
Medium	-0.084 (-0.179, 0.010)	0080	0.166 (-0.038,0.370)	0.111	-0.066 (-0.145,0.014)	0.106	-0.102 (-0.241,0.036)	0.149
High	Ref.		Ref.		Ref.		Ref.	
Father’s educational level								
Primary school and below	-0.014 (-0.166,0.139)	0.859	-0.348 (-0.678, -0.018)	0.039	0.074 (-0.055,0.203)	0.258	-0.105 (-0.330,0.119)	0.357
Middle school	-0.065 (-0.189,0.060)	0.309	-0.291 (-0.561,-0.021)	0.035	0.001 (-0.105,0.106)	0.995	-0.069 (-0.253,0.115)	0.461
Senior high school and above	Ref.		Ref.		Ref.		Ref.	
Mother’s educational level								
Primary school and below	0.069 (-0.088,0.225)	0.389	-0.079 (-0.418,0.260)	0.649	0.054 (-0.079,0.186)	0.427	-0.005 (-0.235,0.225)	0.965
Middle school	0.033 (-0.114,0.180)	0.659	-0.094 (-0.413,0.224)	0.562	0.002 (-0.123,0.126)	0.981	0.007 (-0.209,0.224)	0.947
Senior high school and above	Ref.		Ref.		Ref.		Ref.	
Cigarette use	0.094 (-0.089,0.276)	0.313	-0.260 (-0.655,0.136)	0.198	0.218 (0.064,0.372)	0.006	-0.004 (-0.272,0.265)	0.977
Alcohol use	0.073 (-0.037,0.184)	0.193	-0.030 (-0.269,0.209)	0.807	0.088 (-0.005,0.181)	0.065	-0.135 (-0.298,0.027)	0.103

Inflammatory cytokines were log-transformed before analysis; the crude model was not adjusted by any variables, the adjusted model was adjusted by residential area, self-reported family economy, self-rated health condition, father’s education level, mother’s education level, cigarette use and alcohol use.

B, regression coefficient; CI, confidence interval; IL-1β, interleukin-1β; IL-6, interleukin-6; TNF-α, tumor necrosis factor-α; CRP, C reactive protein.

a Δ represented changes in inflammatory biomarkers levels between 2-year follow-up and baseline.

**Figure 1:**
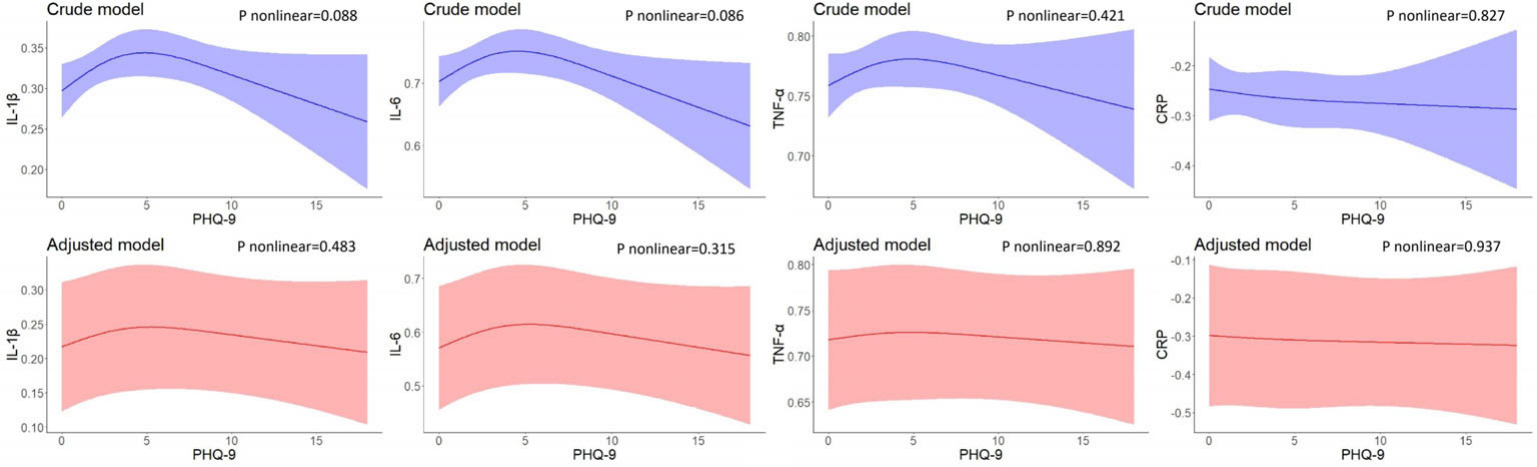
Restricted cubic spline models of the associations between PHQ-9 total scores and inflammatory biomarkers at baseline (n=723). IL-1β, interleukin-1β; IL-6, interleukin-6; TNF-α, tumor necrosis factor-α; CRP, C reactive protein. ^a^ The blue plot represented the crude model which was not adjusted by any variables, and the red plot represented the adjusted model which was adjusted by residential area, self-reported family economy, self-rated health condition, father’s education level, mother’s education level, cigarette use and alcohol use.

**Figure 2:**
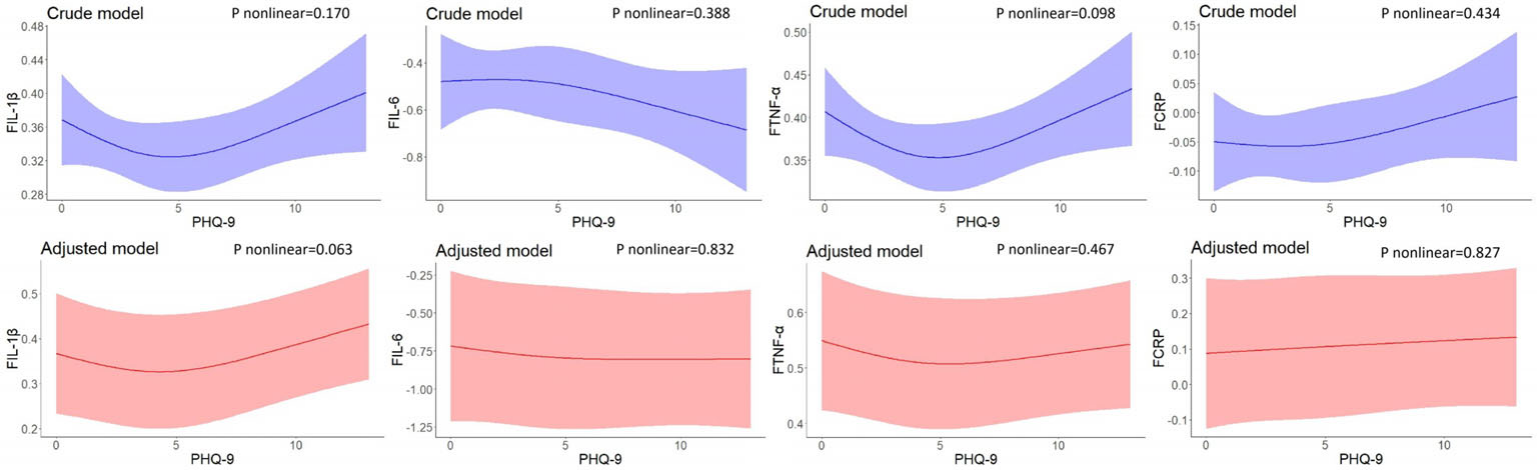
Restricted cubic spline models of the associations between PHQ-9 scores at baseline and inflammatory biomarkers at follow-up (n=248). IL-1β, interleukin-1β; IL-6, interleukin-6; TNF-α, tumor necrosis factor-α; CRP, C reactive protein. ^a^ The blue plot represented the crude model which was not adjusted by any variables, and the red plot represented the adjusted model which was adjusted by residential area, self-reported family economy, self-rated health condition, father’s education level, mother’s education level, cigarette use and alcohol use. ^b^ F represented 2-year follow-up.

**Figure 3:**
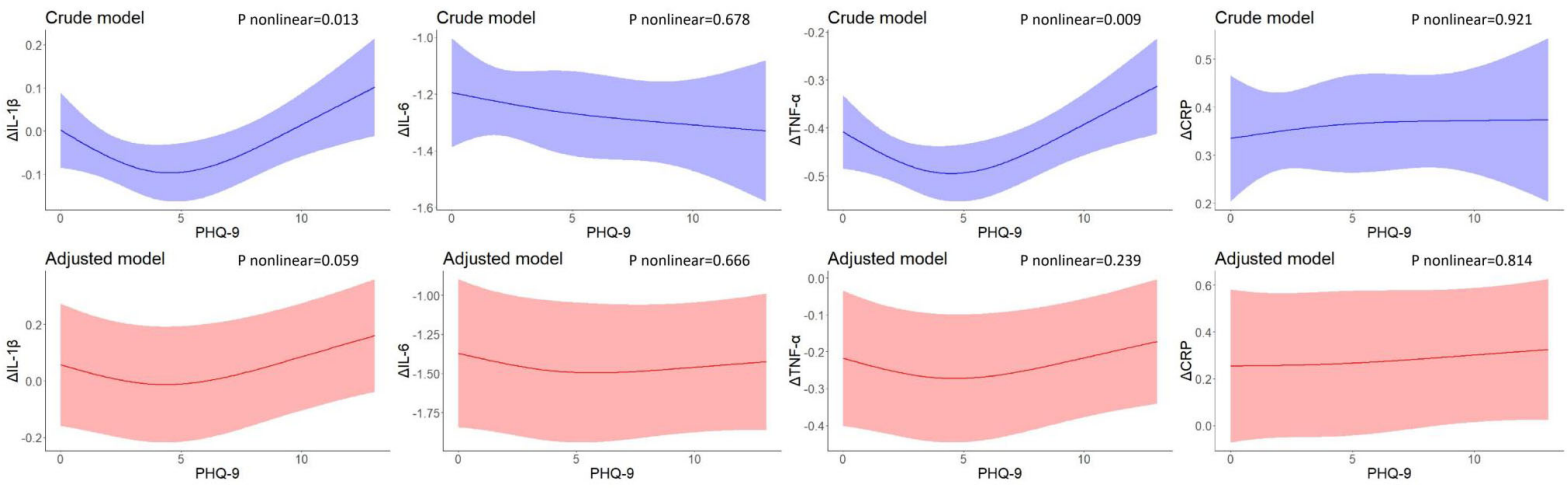
Restricted cubic spline models of the associations between PHQ-9 scores at baseline and changes in inflammatory biomarkers (n=248). IL-1β, interleukin-1β; IL-6, interleukin-6; TNF-α, tumor necrosis factor-α; CRP, C reactive protein. ^a^ The blue plot represented the crude model which was not adjusted by any variables, and the red plot represented the adjusted model which was adjusted by residential area, self-reported family economy, self-rated health condition, father’s education level, mother’s education level, cigarette use and alcohol use. ^b^ Δ represented changes between 2-year follow-up with baseline.

**Figures 1**–**3** illustrate the nonlinear relationship between baseline PHQ-9 scores and inflammatory biomarker levels at baseline or 2-year follow-up, as well as the changes in inflammatory biomarker levels between follow-up and baseline. As illustrated in **Figure 1**, baseline PHQ-9 scores were not found non-linearly associated with baseline inflammatory biomarker levels.

No nonlinear associations were found between PHQ-9 scores at baseline and inflammatory biomarker levels at 2-year follow-up. After adjusting for residential area, self-reported family economy, self-rated health condition, father’s education level, mother’s education level, cigarette use, and alcohol use, the results did not change significantly (**Figure 2**).

**Figure 3** shows the U-shaped nonlinear associations of PHQ-9 scores with changes in IL-1β, IL-6, TNF-α, and CRP levels between 2-year follow-up and baseline. As PHQ-9 scores increased, changes in IL-1β decreased until the PHQ-9 scores were around 4 and increased eventually (*P* nonlinear = 0.013), However, the effects were attenuated and no longer significant after adjusting for covariates (*P* nonlinear = 0.059). Similarly, U-shaped associations were found between baseline PHQ-9 scores and changes in TNF-α levels (*P* nonlinear = 0.009), but no nonlinear associations persisted after controlling for residential area, self-reported family economy, self-rated health condition, father’s education level, mother’s education level, cigarette use, and alcohol use. No associations were found for change in IL-6 and CRP levels between 2-year follow-up and baseline.

### 3.3 Associations between the symptom-specificity of depressive symptoms at baseline and inflammatory biomarkers

**Figure 4:**
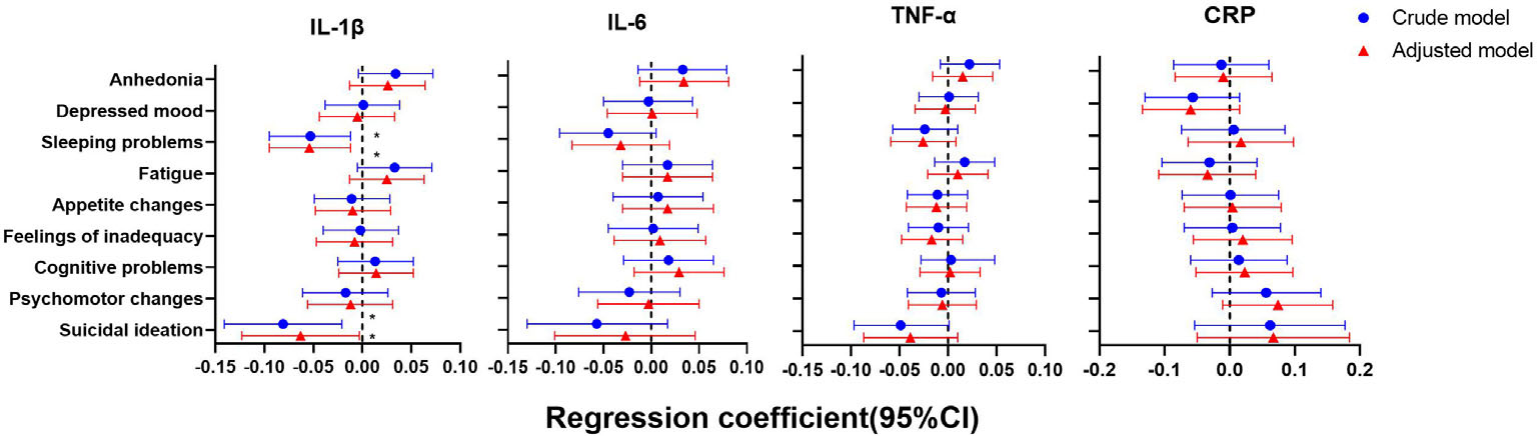
Generalized linear models of the associations between symptom-specificity of depressive symptoms and inflammatory biomarkers at baseline (n=723). IL-1β, interleukin-1β; IL-6, interleukin-6; TNF-α, tumor necrosis factor-α; CRP, C reactive protein. ^a^ The blue plot represented the crude model which was not adjusted by any variables, and the red plot represented the adjusted model which was adjusted by residential area, self-reported family economy, self-rated health condition, father’s education level, mother’s education level, cigarette use and alcohol use.

**Figure 5:**
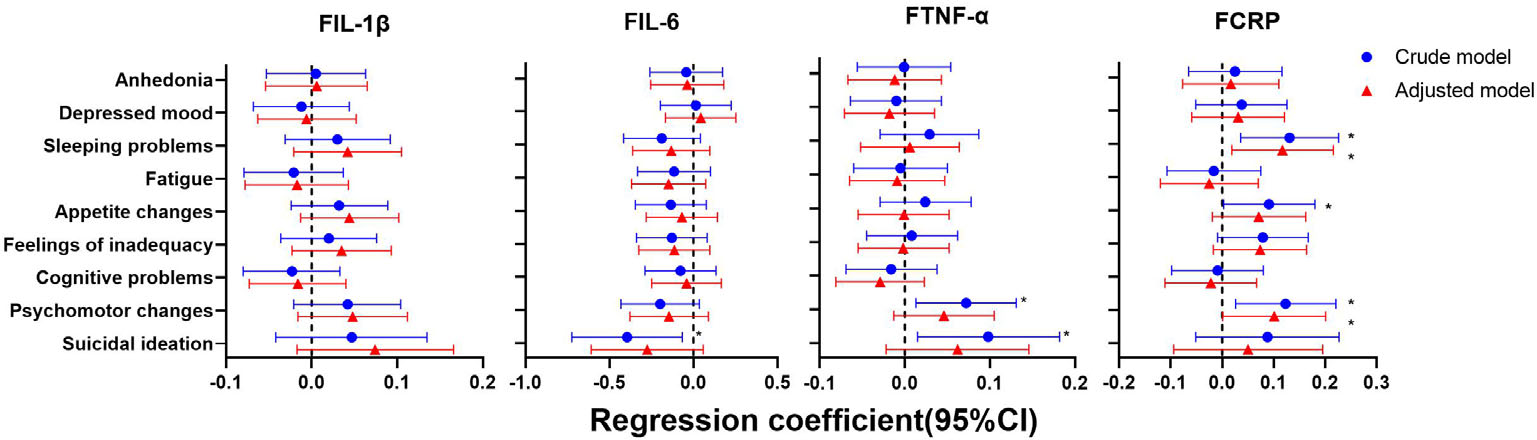
Generalized linear models of the associations between symptom-specificity of depressive symptoms at baseline and inflammatory biomarkers at follow-up (n=248). IL-1β, interleukin-1β; IL-6, interleukin-6; TNF-α, tumor necrosis factor-α; CRP, C reactive protein. ^a^ The blue plot represented the crude model which was not adjusted by any variables, and the red plot represented the adjusted model which was adjusted by residential area, self-reported family economy, self-rated health condition, father’s education level, mother’s education level, cigarette use and alcohol use. ^b^ F represented 2-year follow-up.

**Figure 6:**
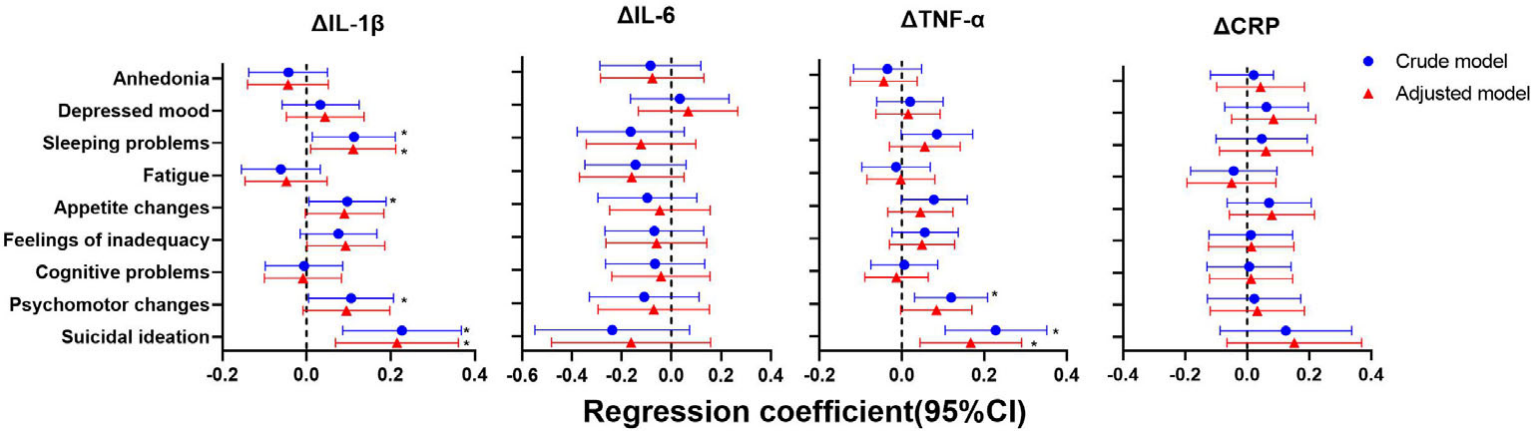
Generalized linear models of the associations between symptom-specificity of depressive symptoms at baseline and changes in inflammatory biomarkers between follow-up and baseline (n=248). IL-1β, interleukin-1β; IL-6, interleukin-6; TNF-α, tumor necrosis factor-α; CRP, C reactive protein. ^a^ The blue plot represented the crude model which was not adjusted by any variables, the red plot represented adjusted model which was adjusted by residential area, self-reported family economy, self-rated health condition, father’s education level, mother’s education level, cigarette use and alcohol use. ^b^ Δ represented changes between 2-year follow-up with baseline.

As shown in **Figures 4**–**6**, after adjustment for residential area, self-reported family economy, self-rated health condition, father’s education level, mother’s education level, cigarette use and alcohol use, sleeping problems, and suicidal ideation were associated with lower IL-1β at baseline. Sleeping problems and psychomotor changes at baseline were associated with higher CRP at 2-year follow-up. Similarly, we also found sleeping problems and suicidal ideation at baseline were associated with changes in IL-1β levels. Suicidal ideation at baseline was associated with changes in TNF-α levels. Other individual depressive symptoms were not associated with these inflammatory biomarker levels.

### 3.4 Sensitivity analysis

In a sensitivity analysis, we compared demographic differences between the dropout and the included samples, and the only statistical difference found was in gender. Compared to participants included in the study sample, participants who were not included were more likely to be females. **Supplementary Table S1** shows detailed general characteristic information.

## 4 Discussion

To our knowledge, this is the first study on simultaneous analysis of linear and nonlinear relationships between depressive symptoms and inflammation at the transition to adulthood by a prospective study design, and further analysis of the associations between symptom-specificity of depressive symptoms and inflammation was performed. Our findings indicated a significant U-shaped nonlinear link between depressive symptoms at baseline and changes in IL-1β and TNF-α levels between 2-year follow-up and baseline, but these associations disappeared after adjusting the confounders. Symptom-specificity of depressive symptoms such as sleeping problems, psychomotor changes, and suicidal ideation were associated with inflammation. This highlights the need for further research into the biological mechanisms of depression in adolescence and the possibility of early intervention in this special population, which may help reduce the risk of adverse outcomes.

The transition from adolescence to adulthood has been seen as a decisive phase where mental health can undergo dramatic changes by changing roles and situations (19). For the reason, of the differences in assessment and research design, the results of the systematic review indicated that the prevalence of depressive symptoms in college students was obviously higher than that of the general population, which reported prevalence ranged from 10% to 85%, with a weighted average prevalence of 30.6%, which was also basically consistent with the 40.1% detection rate of depressive symptoms in our survey (28). In a longitudinal prospective study from childhood to adulthood, up to 72% of patients with major depressive disorder relapsed at intervals of 3-5 years after the first episode (29). In addition, individuals with the onset and recurrent episodes of depression in adolescence represented a particularly severe group and were strongly associated with impairment in multiple psychosocial domains (30). Moreover, early adult life is considered a specific risky age, and research on young adults with depressive symptoms across the transition period was of concern.

We identified a significant nonlinear association between depressive symptoms and changes in inflammatory cytokine TNF-α level between 2 years of follow-up and baseline at the transition to adulthood, in line with a previous systematic review supporting a link between chronic inflammation with depressive symptoms (31). Genome-wide association studies (GWAS) reported so far on depression only found a genetic association of TNF-α with depression (32). Furthermore, animal experimental results showed that TNF-α produced a depression-like state in mice reinforcing the idea that inflammatory components may play a crucial role in the pathophysiology of depressive symptoms (33).

The current study sheds light on a dose-response relationship between depressive symptoms at baseline and changes in inflammatory cytokine IL-1β levels after 2 years of follow-up at the transition to adulthood. A recent study showed the involvement of the IL-1 family in the early stage of depression, especially for pro-inflammatory cytokine IL-1β (34). Another study found that focused interventions for individuals with acute coronary syndrome with high IL-1β levels may reduce the risk of future depression (35). Our findings confirm and extend previous epidemiological studies suggesting that depression could be related to the development of chronic inflammation in young adults.

However, we found no statistical cross-sectional or longitudinal link between depressive symptoms and the inflammatory biomarkers IL-6 and CRP, which may be due to random measurement errors due to its diurnal variation (36). CRP was involved in the acute phase response and was sensitive to short-term effects such as infection, so there may be a mix of chronic and acute effects (16). Consistent with our findings, a recent longitudinal survey also reported no association between depressive symptoms and IL-6 (37). Whether there was an association between depression and inflammation, previous research was also conflicting (38, 39). Another longitudinal study found that more severe depression at baseline predicted higher levels of IL-6 at follow-up (40). Settled levels of inflammatory cytokines such as IL-6 were often interpreted as a chronic, low-grade inflammatory state. It should be noted, however, that IL-6 was not only a pro-inflammatory factor, but they were also anti-inflammatory and play a role in tissue maintenance and repair as well as in immune preparation (6). The inflammatory system has been the focus of treatment trials for depression (41). Nevertheless, not all human beings with depression show increased inflammation. For example, there is great heterogeneity in the presentation of depression symptoms (42).

Our findings support the hypothesis that inflammation may be associated with certain specific symptoms such as sleep problems, suicidal ideation, and psychomotor changes in individuals with depression, which did not change statistically after adjustment for key controlling variables. A recent meta-analytic of 15 population-based cohort studies confirmed the symptom-specificity of the relationship between depression and inflammation (43). A prior study using data from UK Biobank and NESDA cohorts indicated that chronic inflammation was associated with somatic/neurovegetative symptoms of sleeping problems (10). Another research demonstrated chronic inflammation was not strongly linked to overall depression scores or severity, but rather related to specific features of depression marked by eating, appetite, and tiredness (44). The profile of symptoms identified can be used to define the subgroups of depressed individuals most likely to benefit from anti-inflammatory therapy. Our results may have vital implications for future research as it suggests a more targeted, symptom-focused approach to exploring the link between depression and chronic inflammation.

In addition to the causative pathway, depressive symptoms and inflammation may be associated, at least in part, by sharing pathophysiological processes. This was suggested by previous research manifesting that both depression and inflammation are associated with the same genetic variants (45, 46). For example, inflammatory cytokine gene polymorphisms are associated with depression (47). There may be multiple interactions between depression and inflammatory cytokines. Traditionally, the biological mechanisms of depression regulation were complex. The impact of depression on chronic inflammation may be a multifactorial process involving the interaction of multiple mechanisms. More exploration is vital to further figure out these complex connections and to try to clarify their underlying mechanisms. We should focus attention on the need for further investigation and evaluation into the biological mechanisms underlying depressive symptoms among adolescence and propose effective intervention methods. A growing body of research has recently linked an unhealthy lifestyle to depression and inflammation. Hence, lifestyle changes may be effective, low-cost preventive interventions (48, 49).

The sensitivity analysis, comparing the included and excluded samples, illustrate that the included participants were more likely to be males, which may lead to extra caution in the extrapolation of the results. Evidence indicated that depression and IL-6 were more closely correlated in women than in men, which may also lead to bias in our findings (50). Nevertheless, a systematic review suggested that gender differences in the association between depressive symptoms and systemic inflammation were not significant (51). More relevant studies comparing men and women would be required. Besides, because of the potentially confounding effects of the menstrual cycle and estrogen on inflammatory biomarkers, systematic studies targeting women are highly warranted (52, 53).

Several strengths of this study should be addressed. Firstly, this is one of the few studies that comprehensively explore the causal associations linking depressive symptoms and multiple inflammatory biomarkers longitudinally. Most previous research was cross-sectional, our design was a step forward in evaluating the temporal association between depressive symptoms and chronic inflammation. Secondly, this research applied an innovative analytic approach to restricted cubic splines to analyze the nonlinear association of depressive symptoms with inflammation. Conversely, the contributions of this study are required to be considering its limitations in mind. Blood samples were only collected at two points to measure inflammatory biomarker levels, and the duration of follow-up may be a factor in the difference in results. However, no studies have shown how long the effects of depressive symptoms on chronic inflammation will last. Secondly, a large sample size lost to follow-up may lead to biased results, but we conducted a sensitivity analysis of participants who were included and excluded, except for gender, and general characteristics were statistically comparable. Thirdly, depressive symptoms were assessed by self-reporting rather than clinical diagnoses, which may not avoid recall bias and reporting bias. However, studies have found that convenient self-report measures of depression may be more helpful in predicting adverse outcomes than clinical scores (22, 23). Future research would preferably apply the clinical standard for the diagnosis of depressive symptoms.

In summary, our study identified that there was a significant nonlinear relationship between baseline depressive symptoms and changes in IL-1β and TNF-α levels during a 2-year follow-up, but these associations disappeared after adjusting the confounders. Symptom-specificity of depressive symptoms such as sleeping problems, psychomotor changes, and suicidal ideation were associated with inflammation during a 2-year follow-up at the transition to adulthood. Collectively, our results support the effect of depressive symptoms on chronic inflammation, and future research with larger samples and more well-designed waves of follow-up is imperative to verify our findings. Nonetheless, this information could be useful for the early identification of depressive symptoms and may provide clues for new symptom-based psychological and pharmacological treatments for individuals with elevated levels of inflammation.

## Data availability statement

The raw data supporting the conclusions of this article will be made available by the authors, without undue reservation.

## Ethics statement

The studies involving human participants were reviewed and approved by Ethics Committee of Anhui Medical University (No. 20170291). Written informed consent to participate in this study was provided by the participants’ legal guardian/next of kin.

## Author contributions

SZ was responsible for study conceptualization, formal analysis, and writing the original draft of the manuscript. YQ, DZ, TL, YX, LZ, and YY contributed to data collection. XW and ST supervised the study, were responsible for funding acquisition, and reviewed and edited the manuscript. FT contributed to resource acquisition. All authors contributed to the article and approved the submitted version.
