# Supplementary material for: Depressive symptoms predict longitudinal changes of chronic inflammation at the transition to adulthood
Source: Front Immunol. 2023 Jan 4;13:1036739. doi: 10.3389/fimmu.2022.1036739 (PMC9846044; doi:10.3389/fimmu.2022.1036739)
Supplement: Supplementary file 1 [file Table_1.docx]

**Table S1**  Sensitivity analysis.

| Variables | Included (n=248) | Excluded (n=475) | *P* value |
| --- | --- | --- | --- |
| **Gender** |  |  | ＜0.001 |
| Male | 105 (42.3) | 135 (28.2) |  |
| Female | 143 (57.7) | 344 (71.8) |  |
| Age, mean±SD | 18.69±0.97 | 18.67±1.00 | 0.802 |
| BMI, mean±SD | 20.89±2.53 | 20.65±2.62 | 0.230 |
| **Residential area** |  |  | 0.055 |
| Rural | 151 (60.9) | 256 (53.4) |  |
| Urban | 97 (39.1) | 223 (46.6) |  |
| **Any siblings** |  |  | 0.461 |
| No | 59 (23.8) | 126 (26.3) |  |
| Yes | 189 (76.2) | 353 (73.7) |  |
| **Self-reported family economy** |  |  | 0.146 |
| Low | 63 (25.4) | 99 (20.7) |  |
| Medium or high | 185 (74.6) | 380 (79.3) |  |
| **Self-rated health** |  |  | 0.338 |
| Low | 6 (2.4) | 18 (3.8) |  |
| Medium or high | 242 (97.6) | 461 (96.2) |  |
| **Father’s education level** |  |  | 0.812 |
| Primary school and below | 55 (22.2) | 105 (21.9) |  |
| Middle school | 124 (50.0) | 230 (48.0) |  |
| Senior high school and above | 69 (27.8) | 144 (30.1) |  |
| **Mother’s education level** |  |  | 0.234 |
| Primary school and below | 115 (46.4) | 207 (43.2) |  |
| Middle school | 90 (36.3) | 163 (34.0) |  |
| Senior high school and above | 43 (17.3) | 109 (22.8) |  |
| **Cigarette use** |  |  | 0.166 |
| No | 228 (91.9) | 453 (94.6) |  |
| Yes | 20 (8.1) | 26 (5.4) |  |
| **Alcohol use** |  |  | 0.084 |
| No | 185 (74.6) | 384 (80.2) |  |
| Yes | 63 (25.4) | 95 (19.8) |  |
| **Depressive symptoms** |  |  | 0.285 |
| No | 141 (56.9) | 292 (61.0) |  |
| Yes | 107 (43.1) | 187 (39.0) |  |

SD, standard deviation; BMI, body mass index.
