# Supplementary material for: Depressive symptoms predict longitudinal changes of chronic inflammation at the transition to adulthood
Source: Front Immunol. 2023 Jan 4;13:1036739. doi: 10.3389/fimmu.2022.1036739 (PMC9846044; doi:10.3389/fimmu.2022.1036739)
Supplement: Supplementary file 2 [file Table_2.docx]

**Table S2** Generalized linear models of the associations between symptom-specificity of depressive symptoms and inflammatory biomarkers at baseline (n=723).

| Depressive symptoms | IL-1β | |  | IL-6 | |  | TNF-α | |  | CRP | |
| --- | --- | --- | --- | --- | --- | --- | --- | --- | --- | --- | --- |
|  | *B* (95% *CI*) | *P* value |  | *B* (95% *CI*) | *P* value |  | *B* (95% *CI*) | *P* value |  | *B* (95% *CI*) | *P* value |
| **Crude model** |  |  |  |  |  |  |  |  |  |  |  |
| Anhedonia | 0.034 (-0.004,0.072) | 0.080 |  | 0.033 (-0.014,0.079) | 0.166 |  | 0.022 (-0.008,0.053) | 0.154 |  | -0.013 (-0.086,0.060) | 0.734 |
| Depressed mood | 0.001 (-0.038,0.038) | 0.982 |  | -0.003 (-0.050,0.043) | 0.891 |  | 0.001 (-0.030,0.031) | 0.966 |  | -0.057 (-0.130,0.015) | 0.122 |
| Sleeping problems | -0.053 (-0.095，-0.012) | 0.011 |  | -0.045 (-0.096,0.005) | 0.079 |  | -0.024 (-0.057,0.010) | 0.164 |  | 0.006 (-0.074,0.085) | 0.889 |
| Fatigue | 0.033 (-0.005,0.071) | 0.090 |  | 0.017 (-0.030,0.064) | 0.471 |  | 0.017 (-0.014,0.048) | 0.276 |  | -0.031 (-0.104,0.042) | 0.403 |
| Appetite changes | -0.011 (-0.049,0.028) | 0.586 |  | 0.007 (-0.040,0.054) | 0.766 |  | -0.011 (-0.042,0.020) | 0.500 |  | 0.001 (-0.073,0.075) | 0.974 |
| Feelings of inadequacy | -0.002 (-0.040,0.037) | 0.930 |  | 0.002 (-0.045,0.049) | 0.942 |  | -0.010 (-0.041,0.021) | 0.543 |  | 0.004 (-0.070,0.078) | 0.913 |
| Cognitive problems | 0.013 (-0.025,0.052) | 0.499 |  | 0.018 (-0.029,0.065) | 0.451 |  | 0.003 (-0.028,0.048) | 0.827 |  | 0.014 (-0.060,0.088) | 0.716 |
| Psychomotor changes | -0.017 (-0.061,0.026) | 0.435 |  | -0.023 (-0.076,0.030) | 0.394 |  | -0.007 (-0.042,0.028) | 0.684 |  | 0.056 (-0.027,0.140) | 0.186 |
| Suicidal ideation | -0.081 (-0.141，-0.021) | 0.008 |  | -0.057 (-0.130,0.017) | 0.133 |  | -0.049 (-0.097,0.001) | 0.048 |  | 0.062 (-0.054,0.177) | 0.297 |
| **Adjusted model** |  |  |  |  |  |  |  |  |  |  |  |
| Anhedonia | 0.026 (-0.013,0.064) | 0.189 |  | 0.034 (-0.012,0.081) | 0.150 |  | 0.015 (-0.016,0.046) | 0.347 |  | -0.010 (-0.084,0.065) | 0.798 |
| Depressed mood | -0.005 (-0.044,0.033) | 0.783 |  | 0.001 (-0.046,0.048) | 0.969 |  | -0.003 (-0.034,0.028) | 0.844 |  | -0.060 (-0.134,0.015) | 0.115 |
| Sleeping problems | -0.054 (-0.095，-0.012) | 0.011 |  | -0.032 (-0.083,0.019) | 0.220 |  | -0.026 (-0.059,0.008) | 0.135 |  | 0.017 (-0.064,0.098) | 0.679 |
| Fatigue | 0.025 (-0.013,0.063) | 0.202 |  | 0.017 (-0.030,0.064) | 0.470 |  | 0.010 (-0.021,0.041) | 0.539 |  | -0.034 (-0.109,0.040) | 0.365 |
| Appetite changes | -0.010 (-0.048,0.029) | 0.615 |  | 0.017 (-0.030,0.065) | 0.467 |  | -0.012 (-0.043,0.019) | 0.441 |  | 0.004 (-0.070,0.079) | 0.909 |
| Feelings of inadequacy | -0.008 (-0.047,0.031) | 0.678 |  | 0.009 (-0.039,0.057) | 0.702 |  | -0.017 (-0.048,0.015) | 0.297 |  | 0.020 (-0.056,0.096) | 0.610 |
| Cognitive problems | 0.014 (-0.024,0.052) | 0.475 |  | 0.029 (-0.018,0.076) | 0.223 |  | 0.002 (-0.029,0.033) | 0.886 |  | 0.023 (-0.052,0.097) | 0.548 |
| Psychomotor changes | -0.012 (-0.056,0.031) | 0.584 |  | -0.003 (-0.056,0.050) | 0.910 |  | -0.006 (-0.041,0.029) | 0.746 |  | 0.074 (-0.011,0.158) | 0.089 |
| Suicidal ideation | -0.063 (-0.123，-0.003) | 0.040 |  | -0.027 (-0.101,0.046) | 0.466 |  | -0.039 (-0.087,0.010) | 0.119 |  | 0.067 (-0.050,0.184) | 0.261 |

Note: Inflammatory cytokines were log-transformed before analysis; the crude model was not adjusted by any variables, the adjusted model was adjusted by residential area, self-reported family economy, self-rated health condition, father’s education level, mother’s education level, cigarette use and alcohol use.

Abbreviations: B, regression coefficient; CI, confidence interval; IL-1β, interleukin-1β; IL-6, interleukin-6; TNF-α, tumor necrosis factor-α; CRP, C reactive protein.
