# Supplementary material for: Depressive symptoms predict longitudinal changes of chronic inflammation at the transition to adulthood
Source: Front Immunol. 2023 Jan 4;13:1036739. doi: 10.3389/fimmu.2022.1036739 (PMC9846044; doi:10.3389/fimmu.2022.1036739)
Supplement: Supplementary file 3 [file Table_3.docx]

**Table S3** Generalized linear models of the associations between symptom-specificity of depressive symptoms at baseline and inflammatory biomarkers at follow-up (n=248).

| Depressive symptoms | FIL-1β^a^ | |  | FIL-6 | |  | FTNF-α | |  | FCRP | |
| --- | --- | --- | --- | --- | --- | --- | --- | --- | --- | --- | --- |
|  | *B* (95% *CI*) | *P* value |  | *B* (95% *CI*) | *P* value |  | *B* (95% *CI*) | *P* value |  | *B* (95% *CI*) | *P* value |
| **Crude model** |  |  |  |  |  |  |  |  |  |  |  |
| Anhedonia | 0.005 (-0.053,0.063) | 0.870 |  | -0.043 (-0.259,0.173) | 0.698 |  | -0.001 (-0.056,0.054) | 0.980 |  | 0.025 (-0.065,0.116) | 0.583 |
| Depressed mood | -0.012 (-0.068,0.044) | 0.670 |  | 0.014 (-0.197,0.225) | 0.896 |  | -0.010 (-0.064,0.043) | 0.706 |  | 0.038 (-0.051,0.126) | 0.405 |
| Sleeping problems | 0.030 (-0.031,0.092) | 0.332 |  | -0.188 (-0.416,0.041) | 0.108 |  | 0.029 (-0.029,0.087) | 0.327 |  | 0.131 (0.036,0.226) | 0.007 |
| Fatigue | -0.021 (-0.079,0.037) | 0.481 |  | -0.115 (-0.332,0.101) | 0.297 |  | -0.005 (-0.060,0.050) | 0.852 |  | -0.016 (-0.107,0.075) | 0.732 |
| Appetite changes | 0.032 (-0.024,0.089) | 0.260 |  | -0.134 (-0.346,0.077) | 0.212 |  | 0.024 (-0.029,0.078) | 0.373 |  | 0.091 (0.003,0.180) | 0.042 |
| Feelings of inadequacy | 0.020 (-0.036,0.076) | 0.486 |  | -0.128 (-0.338,0.082) | 0.232 |  | 0.008 (-0.045,0.062) | 0.764 |  | 0.079 (-0.009,0.167) | 0.077 |
| Cognitive problems | -0.023 (-0.080,0.033) | 0.419 |  | -0.077 (-0.289,0.134) | 0.473 |  | -0.016 (-0.069,0.038) | 0.569 |  | -0.009 (-0.098,0.080) | 0.846 |
| Psychomotor changes | 0.042 (-0.021,0.104) | 0.189 |  | -0.198 (-0.431,0.035) | 0.096 |  | 0.072 (0.013,0.131) | 0.017 |  | 0.123 (0.026,0.221) | 0.013 |
| Suicidal ideation | 0.047 (-0.042,0.135) | 0.300 |  | -0.395 (-0.723,-0.066) | 0.018 |  | 0.098 (0.015,0.182) | 0.021 |  | 0.088 (-0.051,0.227) | 0.213 |
| **Adjusted model** |  |  |  |  |  |  |  |  |  |  |  |
| Anhedonia | 0.006 (-0.054,0.065) | 0.845 |  | -0.037 (-0.254,0.181) | 0.741 |  | -0.012 (-0.067,0.043) | 0.668 |  | 0.017 (-0.077,0.110) | 0.729 |
| Depressed mood | -0.006 (-0.063,0.052) | 0.850 |  | 0.044 (-0.166,0.253) | 0.682 |  | -0.018 (-0.071,0.035) | 0.507 |  | 0.031 (-0.059,0.121) | 0.503 |
| Sleeping problems | 0.042 (-0.021,0.105) | 0.189 |  | -0.132 (-0.362,0.098) | 0.261 |  | 0.006 (-0.052,0.064) | 0.833 |  | 0.117 (0.019,0.216) | 0.020 |
| Fatigue | -0.017 (-0.078,0.043) | 0.579 |  | -0.148 (-0.368,0.073) | 0.189 |  | -0.009 (-0.065,0.047) | 0.755 |  | -0.025 (-0.120,0.070) | 0.610 |
| Appetite changes | 0.044 (-0.013,0.102) | 0.133 |  | -0.069 (-0.281,0.143) | 0.521 |  | -0.001 (-0.055,0.052) | 0.957 |  | 0.071 (-0.019,0.162) | 0.124 |
| Feelings of inadequacy | 0.035 (-0.023,0.093) | 0.234 |  | -0.114 (-0.325,0.097) | 0.290 |  | -0.002 (-0.055,0.052) | 0.953 |  | 0.074 (-0.017,0.164) | 0.112 |
| Cognitive problems | -0.016 (-0.073,0.040) | 0.570 |  | -0.041 (-0.248,0.166) | 0.697 |  | -0.029 (-0.081,0.023) | 0.273 |  | -0.022 (-0.111,0.067) | 0.635 |
| Psychomotor changes | 0.048 (-0.016,0.112) | 0.141 |  | -0.145 (-0.378,0.089) | 0.225 |  | 0.046 (-0.013,0.105) | 0.124 |  | 0.101 (0.001,0.201) | 0.049 |
| Suicidal ideation | 0.074 (-0.017,0.166) | 0.110 |  | -0.275 (-0.609,0.058) | 0.106 |  | 0.062 (-0.022,0.146) | 0.149 |  | 0.050 (-0.094,0.195) | 0.495 |

Note: Inflammatory cytokines were log-transformed before analysis; the crude model was not adjusted by any variables, the adjusted model was adjusted by residential area, self-reported family economy, self-rated health condition, father’s education level, mother’s education level, cigarette use and alcohol use.

Abbreviations: B, regression coefficient; CI, confidence interval; IL-1β, interleukin-1β; IL-6, interleukin-6; TNF-α, tumor necrosis factor-α; CRP, C reactive protein.

^a^F represented 2-year follow-up.
