# Supplementary material for: Depressive symptoms predict longitudinal changes of chronic inflammation at the transition to adulthood
Source: Front Immunol. 2023 Jan 4;13:1036739. doi: 10.3389/fimmu.2022.1036739 (PMC9846044; doi:10.3389/fimmu.2022.1036739)
Supplement: Supplementary file 4 [file Table_4.docx]

**Table S4** Generalized linear models of the associations between symptom-specificity of depressive symptoms at baseline and changes in inflammatory biomarkers between follow-up and baseline (n=248).

| Depressive symptoms | ΔIL-1β^a^ | |  | ΔIL-6 | |  | ΔTNF-α | |  | ΔCRP | |
| --- | --- | --- | --- | --- | --- | --- | --- | --- | --- | --- | --- |
|  | *B* (95% *CI*) | *P* value |  | *B* (95% *CI*) | *P* value |  | *B* (95% *CI*) | *P* value |  | *B* (95% *CI*) | *P* value |
| **Crude model** |  |  |  |  |  |  |  |  |  |  |  |
| Anhedonia | -0.043 (-0.137,0.050) | 0.365 |  | -0.084 (-0.288,0.119) | 0.416 |  | -0.035 (-0.117,0.048) | 0.412 |  | 0.021 (-0.118,0.085) | 0.770 |
| Depressed mood | 0.033 (-0.058,0.125) | 0.472 |  | 0.034 (-0.165,0.232) | 0.739 |  | 0.020 (-0.061,0.100) | 0.629 |  | 0.062 (-0.072,0.197) | 0.363 |
| Sleeping problems | 0.113 (0.014,0.211) | 0.025 |  | -0.164 (-0.379,0.052) | 0.137 |  | 0.085 (-0.002,0.172) | 0.055 |  | 0.047 (-0.100,0.194) | 0.530 |
| Fatigue | -0.061 (-0.155,0.033) | 0.206 |  | -0.144 (-0.348,0.059) | 0.165 |  | -0.014 (-0.097,0.069) | 0.745 |  | -0.043 (-0.182,0.096) | 0.547 |
| Appetite changes | 0.097 (0.006,0.189) | 0.036 |  | -0.097 (-0.296,0.102) | 0.339 |  | 0.078 (-0.002,0.159) | 0.056 |  | 0.071 (-0.064,0.207) | 0.302 |
| Feelings of inadequacy | 0.076 (-0.015,0.167) | 0.103 |  | -0.069 (-0.267,0.130) | 0.498 |  | 0.056 (-0.024,0.137) | 0.168 |  | 0.012 (-0.123,0.147) | 0.866 |
| Cognitive problems | -0.006 (-0.098,0.086) | 0.901 |  | -0.066 (-0.265,0.134) | 0.519 |  | 0.006 (-0.075,0.087) | 0.888 |  | 0.007 (-0.129,0.142) | 0.924 |
| Psychomotor changes | 0.106 (0.005,0.207) | 0.040 |  | -0.109 (-0.330,0.111) | 0.332 |  | 0.120 (0.031,0.208) | 0.008 |  | 0.023 (-0.128,0.173) | 0.769 |
| Suicidal ideation | 0.227 (0.086,0.368) | 0.002 |  | -0.238 (-0.549,0.073) | 0.134 |  | 0.228 (0.105,0.352) | <0.001 |  | 0.125 (-0.087,0.337) | 0.249 |
| **Adjusted model** |  |  |  |  |  |  |  |  |  |  |  |
| Anhedonia | -0.044 (-0.140,0.052) | 0.372 |  | -0.077 (-0.285,0.131) | 0.466 |  | -0.044 (-0.125,0.037) | 0.286 |  | 0.043 (-0.098,0.185) | 0.547 |
| Depressed mood | 0.044 (-0.048,0.137) | 0.351 |  | 0.067 (-0.133,0.267) | 0.510 |  | 0.015 (-0.063,0.093) | 0.710 |  | 0.085 (-0.050,0.221) | 0.218 |
| Sleeping problems | 0.111 (0.010,0.212) | 0.032 |  | -0.122 (-0.342,0.098) | 0.276 |  | 0.056 (-0.030,0.142) | 0.201 |  | 0.061 (-0.089,0.211) | 0.424 |
| Fatigue | -0.048 (-0.146,0.049) | 0.333 |  | -0.160 (-0.370,0.051) | 0.137 |  | -0.003 (-0.085,0.080) | 0.946 |  | -0.050 (-0.194,0.093) | 0.494 |
| Appetite changes | 0.090 (-0.003,0.184) | 0.057 |  | -0.047 (-0.249,0.156) | 0.651 |  | 0.045 (-0.034,0.124) | 0.268 |  | 0.080 (-0.057,0.217) | 0.253 |
| Feelings of inadequacy | 0.093 (0.001,0.186) | 0.050 |  | -0.060 (-0.263,0.142) | 0.559 |  | 0.049 (-0.030,0.128) | 0.222 |  | 0.014 (-0.124,0.151) | 0.846 |
| Cognitive problems | -0.008 (-0.100,0.083) | 0.858 |  | -0.042 (-0.240,0.155) | 0.675 |  | -0.013 (-0.090,0.064) | 0.738 |  | 0.013 (-0.121,0.147) | 0.850 |
| Psychomotor changes | 0.095 (-0.008,0.198) | 0.070 |  | -0.071 (-0.295,0.153) | 0.536 |  | 0.084 (-0.003,0.170) | 0.059 |  | 0.033 (-0.119,0.185) | 0.671 |
| Suicidal ideation | 0.215 (0.069,0.361) | 0.004 |  | -0.162 (-0.482,0.158) | 0.321 |  | 0.167 (0.044,0.291) | 0.008 |  | 0.152 (-0.065,0.369) | 0.169 |

Note: Inflammatory cytokines were log-transformed before analysis; the crude model was not adjusted by any variables, the adjusted model was adjusted by residential area, self-reported family economy, self-rated health, father’s education level, mother’s education level, cigarette use and alcohol use.

Abbreviations: B, regression coefficient; CI, confidence interval; IL-1β, interleukin-1β; IL-6, interleukin-6; TNF-α, tumor necrosis factor-α; CRP, C reactive protein.

^a^Δ represented changes in inflammatory biomarkers levels between 2-year follow-up and baseline.
