# Supplementary material for: Depressive symptoms predict longitudinal changes of chronic inflammation at the transition to adulthood
Source: Front Immunol. 2023 Jan 4;13:1036739. doi: 10.3389/fimmu.2022.1036739 (PMC9846044; doi:10.3389/fimmu.2022.1036739)
Supplement: Supplementary file 5 [file Table_5.docx]

**Table S5** Generalized linear models of inflammatory biomarkers and depressive symptoms at follow-up (n=248).

| **Inflammatory biomarkers** | **Crude model** | |  | **Adjusted model** | |
| --- | --- | --- | --- | --- | --- |
|  | ***OR* (95% *CI*)** | ***P* value** |  | ***OR* (95% *CI*)** | ***P* value** |
| **Baseline** |  |  |  |  |  |
| IL-1β | 0.530 (0.186,1.510) | 0.235 |  | 0.548 (0.175,1.717) | 0.302 |
| IL-6 | 0.765 (0.313,1.871) | 0.557 |  | 0.939 (0.367,2.406) | 0.896 |
| TNF-α | 0.633 (0.153,2.628) | 0.529 |  | 0.733 (0.160,3.365) | 0.689 |
| CRP | 0.682 (0.404,1.150) | 0.151 |  | 0.638 (0.364,1.119) | 0.117 |
| **2-year follow-up** |  |  |  |  |  |
| F-IL-1β^a^ | 0.470 (0.108,2.045) | 0.470 |  | 0.570 (0.123,2.645) | 0.473 |
| F-IL-6 | 0.696 (0.496,0.977) | 0.036 |  | 0.692 (0.477,1.004) | 0.052 |
| F-TNF-α | 0.628 (0.158,2.492) | 0.508 |  | 0.473 (0.102,2.185) | 0.338 |
| F-CRP | 0.736 (0.321,1.689) | 0.469 |  | 0.733 (0.305,1.761) | 0.487 |
| **Changes between 2-year follow-up and baseline** | |  |  |  |  |
| ΔIL-1β^b^ | 1.124 (0.509,2.479) | 0.773 |  | 1.176 (0.493,2.806) | 0.715 |
| ΔIL-6 | 0.692 (0.481,0.996) | 0.047 |  | 0.671 (0.453,0.996) | 0.047 |
| ΔTNF-α | 0.983 (0.397,2.434) | 0.971 |  | 0.820 (0.298,2.258) | 0.701 |
| ΔCRP | 1.362 (0.775,2.393) | 0.283 |  | 1.460 (0.799,2.669) | 0.219 |

Note: Inflammatory cytokines were log-transformed before analysis; the crude model was not adjusted by any variables, the adjusted model was adjusted by residential area, self-reported family economy, self-rated health, father’s education level, mother’s education level, cigarette use and alcohol use.

Abbreviations: OR, odds ratio; CI, confidence interval; IL-1β, interleukin-1β; IL-6, interleukin-6; TNF-α, tumor necrosis factor-α; CRP, C reactive protein.

^a^F represented 2-year follow-up with baseline.

^b^Δ represented changes in inflammatory biomarkers levels between 2-year follow-up and baseline.
